# Gel Cleaning in Heritage: Comparison of the Water Release among Gels and Traditional Pads

**DOI:** 10.3390/gels10110708

**Published:** 2024-11-02

**Authors:** Antonio Sansonetti, Cristiano Riminesi, Sónia Mironiouk, Noemi Proietti, Valeria Di Tullio, Roberto Nisticò, Barbara Sacchi, Carmen Canevali

**Affiliations:** 1 Institute for Heritage Science, National Research Council, ISPC—CNR Milan Unit, 20154 Milan, Italy; cristiano.riminesi@cnr.it (C.R.); noemi.proietti@cnr.it (N.P.); valeria.ditullio@cnr.it (V.D.T.); barbara.sacchi@cnr.it (B.S.); 2 Escola Superior de Conservació i Restauració de Béns Culturals de Catalunya (ESCRBCC), 08033 Barcelona, Spain; soniamirdob@gmail.com; 3 Department of Materials Science, University of Milano-Bicocca, INSTM, Via R. Cozzi 55, 20125 Milano, Italy; roberto.nistico@unimib.it (R.N.); carmen.canevali@unimib.it (C.C.)

**Keywords:** gel cleaning, water release, agar gel, gravimetry, heritage, water-sensitive materials

## Abstract

Water release is a crucial aspect when considering cleaning effects on water-sensitive materials. In conservation practice, a water-based cleaning method which limits water release is very often needed. Unfortunately, this is not accompanied by an appropriate measure of the effectively released water. In this paper, water release has been measured by comparing traditional cleaning formulations, such as paper pulp and sepiolite, with several gar gel formulations, used by both Italian and European conservators. The assessment has been carried out by the gravimetric method, using three different stone material specimens as reference: Noto calcarenite, Manciano sandstone and Black Bergamo limestone, whose porosity values and distributions are known. Moreover, water distribution has been evaluated by portable NMR tests. Different commercial agar gel products (Bresciani, CTS, Sigma), having different concentrations (3, 4, and 5%), application modes (rigid at room T or fluid warm gels, with and without inserting Japanese tissue paper), and geometry (horizontal in gravity force direction or vertical), have been compared to obtain a full scenario among different water release mechanisms present in real conservation works. The paper faces the important issue of preparing reproducible chemical or water pads as well, useful for further research aimed at comparing cleaning effects in heritage conservation. The most interesting quantitative results can be summarized as follows. The water release measured from paper pulp and sepiolite was found to be 2 to 4 times higher than from any tested agar gel. Water release decreases by increasing agar concentration; an increase in the agar concentration by 1% induces a decrease in water release in the range 16.98–66.88 g depending on the stone; the increase from 4% to 5% is more obvious with respect to that from 3% to 4%. It is possible to assess the effect of the presence of Japanese paper, which is able to reduce the water release from 18 to 76%, depending on the stone and on the agar used. The gravimetric results were also used in the preliminary calibration tests of a contact probe named System Unit Salinity Index (SUSI), recently patented and useful in providing humidity and salinity indexes in a given porous material.

## 1. Introduction

Cleaning the surface of a heritage object is a very delicate and irreversible work; it is aimed at selectively removing only those substances which endanger the appropriate conservation of the object material while hampering the correct legibility of the object’s cultural values [1,2,3]. Conservation scientists should support conservators to implement cleaning methods to improve their effectiveness and to minimize the possible side effects. For these reasons cleaning operations should be cautious, gradual, selective, and safe for the operator, for the environment, and, of course, for the object itself. Cleaning is planned for removing very different materials from a compositional point of view, considering also the degree of adhesion and internal cohesion. Deposits, metal compound stains, biological growth, and black crusts are only some of many examples. The soiling can be formed directly on the surface, being produced by chemical reactions with some substances coming from the environment (new formation products), or they can be of exogenic origin and simply deposited on the surface. Many other possible mechanisms explain the presence of substances partially or completely extraneous to the original object’s material, but a complete discussion of the topic is out of the scope of the present paper [4]. Anyway, it is worth remembering that solubility plays a crucial role in chemical mechanisms of soiling removal. For this reason, water can be applied to the outer surface of heritage objects via spray, nebulizing systems, pads, or gels. Chemical removal can be implemented by adding organic solvents [5,6], chelant substances [7,8], or enzymes [9,10] mixed in the formulate designed for cleaning. In some restoration works the use of free water-based formulation is discouraged because of the sensitivity of the constituting materials. Hence water release control, when cleaning with a water-based system on a heritage surface, is a crucial point. The scientific literature has covered this issue [11,12], but a precise measure of the amount of water released on reference heritage materials is still lacking.

Traditional pads are usually prepared by mixing water with a thickener: clays, cellulose fibers, wood, or paper pulp. The chemical–physical characteristics of the system allow the water, which is essentially free as to transport phenomena, to be released onto the substrate as a function of its porous microstructure [13].

The phenomena of water release/water absorption are determined by:

(a) thickener’s chemical composition and hydrophilicity;
(b) microstructure of the specific materials used;
(c) procedure of pad making;
(d) porous system of the material receiving the water released. A set of three different stone materials have been chosen and studied in previous published research [14,15].

Hence water release is proportional to the total porosity of the substrate and to its pore size distribution. In other words, it is possible to say that water release and absorption capacity are the two faces of the same coin. Water pads are frequently used as cleaning materials when soluble salts are the main components of efflorescences or concretions. These traditional pads are easy to apply even on vertical surfaces or in undercuts.

In the last two decades gel-forming natural and synthetic polymers have been used to improve cleaning chemical effectiveness, while controlling the water release to the porous substrate [16,17,18]. In fact, in gels the presence of water physically bound to the polymer structure has been proved [19]; the state of physically bounded water should limit the water release.

Agar is a gelling material composed of polysaccharides extracted from red seaweeds (genera Gelidium and Gracilariales) [20]; it can form semi-rigid, physical, thermo-reversible gels by dispersing agar raw powder in water, heating the blend, and cooling at room temperature [21]. At the end of this procedure, the polymer chains form a three-dimensional network containing liquid water in the cavities. Conservators are increasingly appreciating this kind of material for its ability to control the water release, especially when used on water-sensitive materials such as gypsum stuccoworks, mural paintings [22,23], and paper [24].

The article aims to address the following research issues, having significant implications and impacts on the practical implementation of cleaning works and on the safeguarding of water-sensitive heritage surfaces: quantitative comparison of water release among traditional pads and gels, among different commercial agar gels (Bresciani, CTS, and Sigma), between rigid at room T and warm fluid gels, and between horizontal and vertical geometry; evaluation of the effect of Japanese paper and of complex mixtures including sand.

These comparisons are made by very simple gravimetric measures and they have been carried out for the first time to the authors’ knowledge; the study’s focus, the issue of preparing traditional pads with high reproducibility, will be discussed together with its limits. Moreover, stones with a different porous system are proposed as reference set materials, useful to carry out an accurate comparison study and able to work as well in similar future research.

The data obtained from gravimetric measurements were used for a preliminary comparison with other types of data obtained through innovative instrumental systems aimed at studying the water distribution inside the stone porous system by means of:

1. the so-called System Unit Salinity Index (SUSI) probe useful for evaluating the moisture content (MC) in a semi-sphere of about 2 cm in diameter;
2. a portable NMR device connected to a single-sided sensor.

## 2. Results and Discussion

### 2.1. Stone Porous System

The porous system of the three tested stones is given by the total open porosity P% (total volume of the pores in %) and by the distribution of the pore volume, classified according to the pore size [14,25,26].

Noto calcarenite is characterized by a high porosity (36.2%), due to a great amount of macropores (86.8%); the most representative pore class corresponds to a diameter in the range 0.5–5.0 μm [27] (see Table 1 and Table 2). For these reasons the stone has a high water absorption coefficient [14].

Manciano sandstone porosity could be considered intermediate in the range of the tested stones (10.7%) and it is composed of mega- (25.9) and macropores (67.0), having size ranging from 0.01 and 5.0 μm [28]; this stone has a high water absorption coefficient as well.

On the contrary, Bergamo Black limestone has a very low total open porosity (0.5%), quite different from the other limestones; pore size ranges mainly from 1.0 to 4.0 μm, being 12.8% megapores and 67.4% macropores [29] (see Table 1 and Table 2).

Thus, it is possible to observe that the three stones are characterized by a very different total open porosity percentages; moreover, the larger class of pores is macropores for each stone.

### 2.2. Water Release: Comparison among Traditional Pads and Agar Gels

The phenomenon under study could be observed as the water released by pads and gels and the water absorbed by the stone capillary system. In the following series of histograms, the water release, measured via gravimetry, has been displayed. Some considerations can be detailed observing the results.

**Figure 1:**
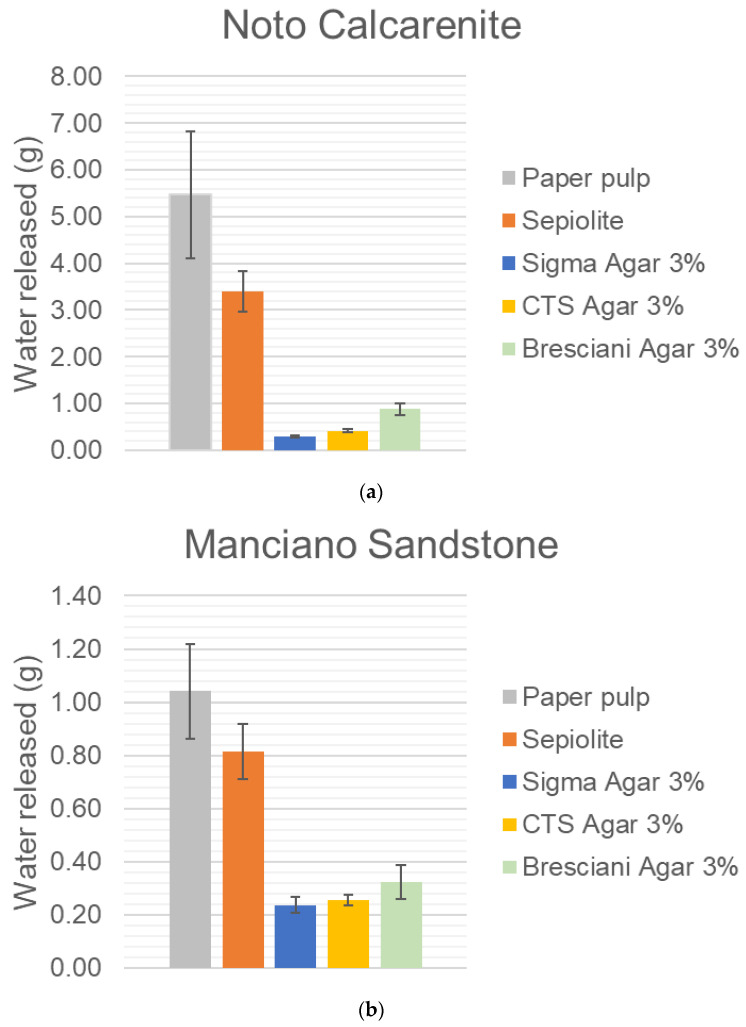

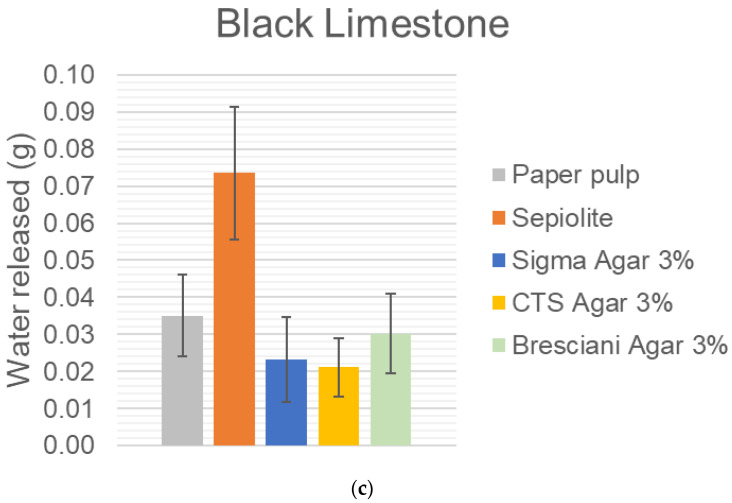
(**a**) Water released on Noto calcarenite. (**b**) Water released on Manciano sandstone. (**c**) Water released on Bergamo Black limestone.

The release of water from paper pulp and sepiolite is greater than from the 3% gels by a factor varying from approximately 2 to 5. This ratio is evident when observing Figure 1a,b, displaying the water releases measured on Noto calcarenite and Manciano sandstone. On the contrary, on the black limestone the reading is heavily affected by experimental error (Figure 1c).

The two lithotypes with the greatest total open porosity (Noto and Manciano; see Table 1) indicate the following trend for the amount of water released by gels:

Noto: Sigma < CTS < Bresciani;
Manciano: Sigma ≈ CTS ≤ Bresciani.

This trend is in agreement with the better cleaning effectiveness observed when gels are used for removing copper stains from marble [30,31].

**Figure 2:**
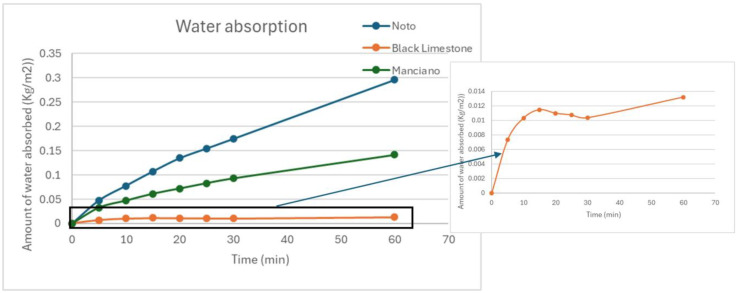
Amount of water absorbed for surface unit vs. time in min (CTS Agar 3% on Noto calcarenite, Manciano sandstone, and Black limestone).

Noto calcarenite, due to its porous structure, absorbs approximately 5 times more water than the Manciano sandstone and about 150 times more than black limestone. In any tested set of specimens, the black Bergamo limestone should not be considered a reliable type of stone to be used in such a procedure. This result is probably due to the very low porosity, the small amount of water absorbed, and hence the high experimental errors; these results are also confirmed by the trends of the water released vs. time, available for the agar CTS gel only. In fact, these curves show that Noto and Manciano absorb the water released by the gel in a gradual and progressive manner (see Figure 2), while the black limestone absorbs in a less regular way and, indeed, in some moments, it seems to release the absorbed water. Moreover, for Noto and Manciano stones it was possible to obtain the absorption coefficient from the curve, while this was not possible for the black limestone due to the non-linear trend (see Table 3).

Paper pulp seems to release a slightly greater amount of water with respect to sepiolite.

**Figure 3:**
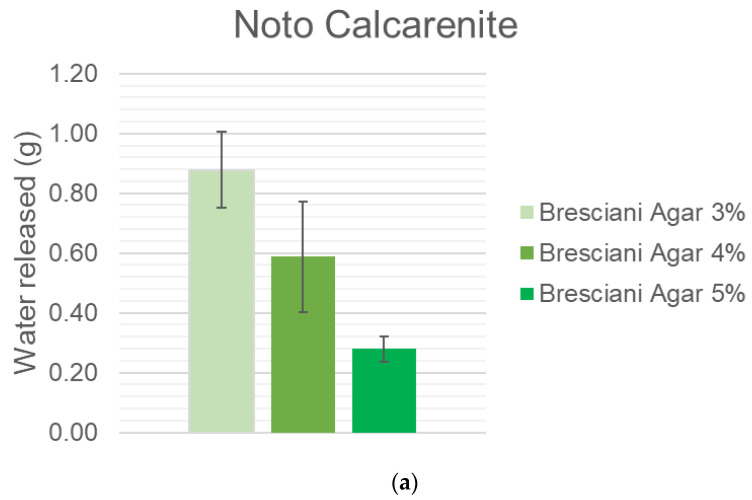

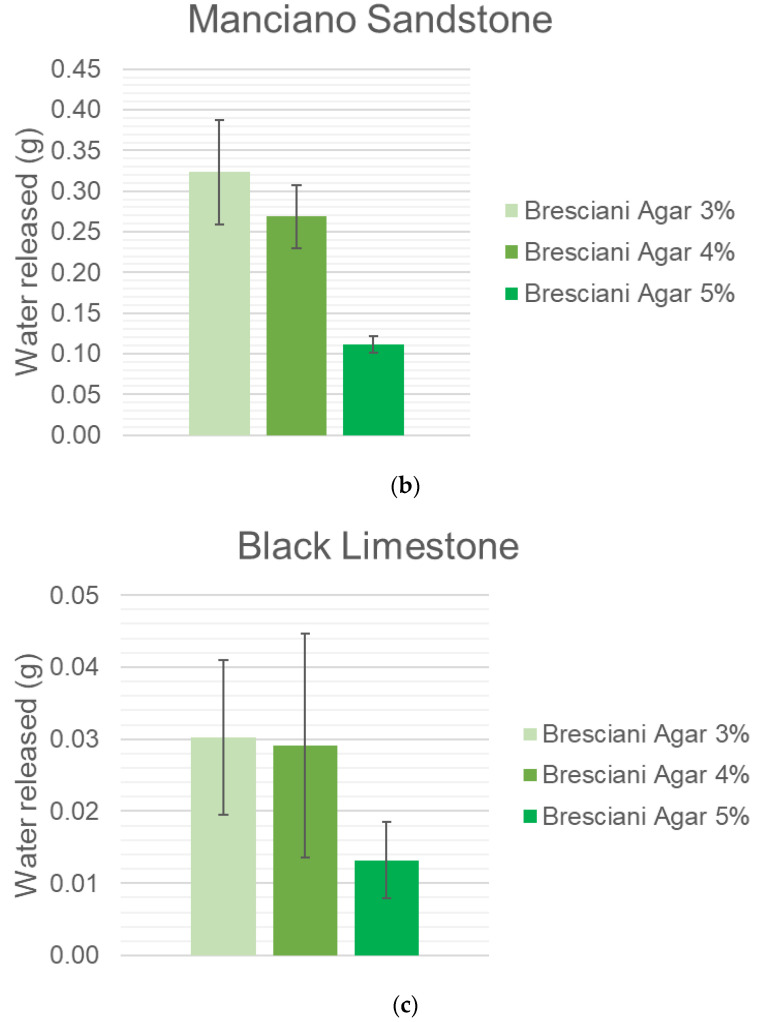
(**a**) Water released on Noto Calcarenite by gels as a function of Bresciani Agar percentage (3%, 4%, 5%). (**b**) Water released on Manciano Sandstone by gels as a function of Bresciani Agar percentage (3%, 4%, 5%). (**c**) Water released on Black limestone by Gels as a function of Bresciani Agar percentage (3%, 4%, 5%).

For what concerns gels, the water release is a linear function of agar concentration (see Figure 3a–c). By increasing the agar concentration from 3% to 5%, the water release lowers by a third; in particular, an increase of 1% in the agar concentration induces a decrease in water release in the range 16.98–66.88 g depending on the stone: the increase from 4% to 5% is more obvious with respect to the one from 3% to 4%; this effect could be due to the greater hampering of the polymeric chains, influencing the water transport. This consideration is evident when looking at Noto and Manciano. Once again, the results of the very low porosity black limestone should not be considered reliable.

**Figure 4:**
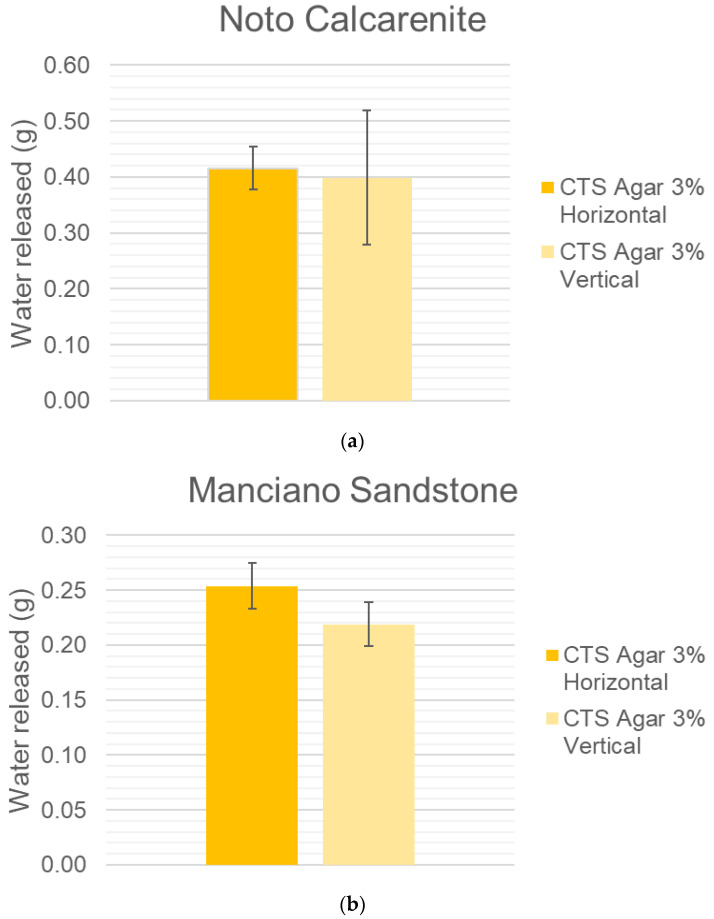
(**a**) Noto calcarenite water release in vertical and horizontal geometry by CTS Agar 3% gel. (**b**) Manciano sandstone water release in vertical and horizontal geometry by CTS Agar 3% gel.

No visible effect was observed between the horizontal geometry to the vertical one (see Figure 4a,b). Hence, the overall experimentation results, more easily obtained in the laboratory for the horizontal geometry, can be translated to conservation sites, where usually the water is released via a vertical geometry.

**Figure 5:**
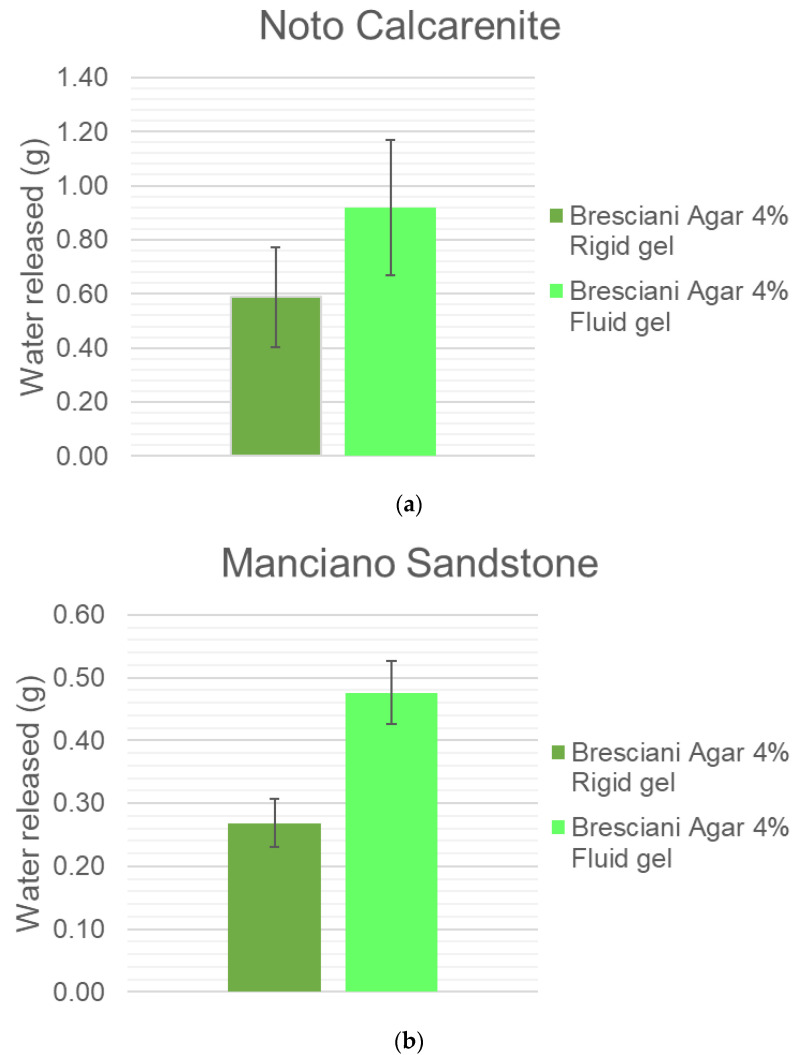
(**a**) Noto calcarenite water release by Bresciani Agar 4%, applied as warm fluid and as rigid gel. (**b**) Manciano sandstone water release by Bresciani Agar 4%, applied as warm fluid and as rigid gel.

When agar is used as a warm fluid at around 40–45 °C, it allows a greater water release than as rigid gels at room temperature (see Figure 5a,b); once again the trend is evident in Noto and Manciano, but not in black limestone.

**Figure 6:**
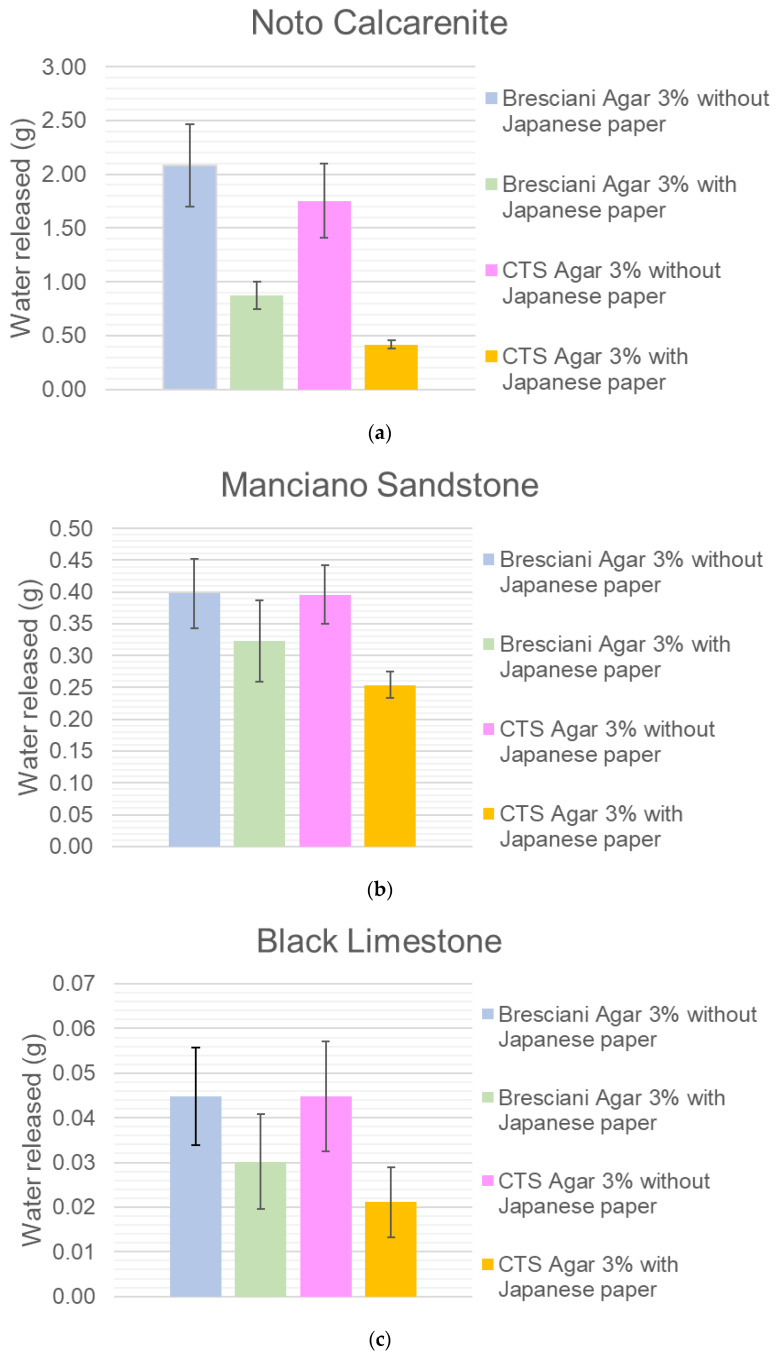
(**a**) Noto calcarenite. Comparison of water release with and without the Japanese paper. CTS Agar 3% and Bresciani Agar 3%. (**b**) Manciano sandstone. Comparison of water release with and without the Japanese paper. CTS Agar 3% and Bresciani Agar 3%. (**c**) Black limestone. Comparison of water release with and without the Japanese paper. CTS Agar 3% and Bresciani Agar 3%.

For what concerns the role of Japanese paper, there is a sort of “barrier effect” by the Japanese paper, more evident for Noto stone. In fact, looking at the histograms in Figure 6a,b, it is possible to assess the effect of the presence of Japanese paper, which brings a heavy reduction of the water release from 18 to 76%, depending on the stone and on the agar used. Bresciani agar is more sensitive to the presence of Japanese paper.

Finally, black limestone does not allow any reliable consideration (Figure 6c).

### 2.3. Water Release and Distribution as Analyzed by Portable NMR

**Figure 7:**
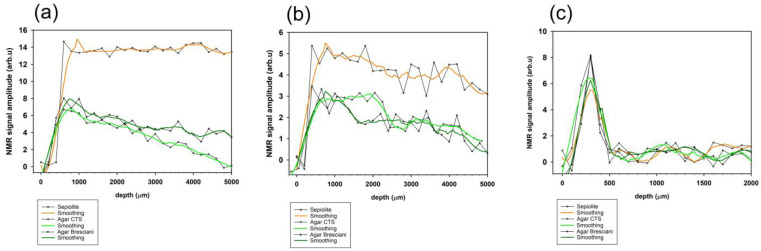
¹H NMR depth profiles for Noto stone (**a**), Manciano stone (**b**), and Black limestone (**c**) after the application of Sepiolite, Agar CTS, and Agar Bresciani at 3% concentration for 30 min.

The amplitude of the NMR signal corresponds to the concentration of water at different depths. Higher amplitudes indicate greater water content, while lower amplitudes suggest less or no water. By scanning at different depths, NMR can reveal how water is distributed from the surface into the interior of the material. This helps in understanding the extent of water penetration and how it varies with depth. The shape of the depth profile can provide insights into the material’s absorption characteristics. For instance, a sharp increase in signal near the surface followed by a plateau suggests surface absorption with limited deeper penetration. Conversely, a gradual increase in signal depth indicates more uniform water distribution. Furthermore, the maximum depth at which water penetration is detected can be determined by analyzing where the NMR signal starts to diminish or disappears. This provides an estimate of how deeply water can infiltrate the material over a given time period. In Figure 7, all the 1H depth profiles acquired in Noto, Manciano, and Bergamo black limestone are shown.

¹H depth profiles acquired in Noto stones (Figure 7a) reveal that Noto treated with sepiolite exhibits the highest NMR signal amplitude, with a sharp rise near the surface (0–1000 µm) followed by a plateau as depth increases (up to 5000 µm). Noto stones treated by CTS agar and Bresciani agar show overall lower NMR signal amplitudes, with a gradual increase in the first 1000 μm and a gradual decline in signal as depth increases. The maximum penetration depth of water into the stone also shows significant differences among sepiolite, Bresciani agar, and CTS agar. In CTS agar, the ¹H NMR signal decreases to zero, indicating that water penetration does not reach a depth of 5 mm within the stone after 30 min of application. Bresciani agar releases a larger amount of water at deeper depths compared to CTS agar.

In Manciano stone (Figure 7b), ^1^H depth profiles follow a similar pattern, though the NMR signal amplitudes are lower overall compared to (a). Sepiolite again shows the highest signal, but the profiles of CTS agar and Bresciani agar are closer in magnitude to one another. Agar gels show a greater variation in the signal across the depth, particularly in the first 1000–2000 µm, followed by a gradual decline.

In black limestone (Figure 7c), the depth profiles show significantly lower NMR signal amplitudes, suggesting no water absorption within the porous structure. All three gels exhibit similar profiles, with initial peaks in the first 500 µm, followed by a drop-off as depth increases. This indicates that water molecules are distributed in a thin layer on the surface of the stone. Overall, these profiles illustrate how the different gels interact with various substrates in terms of water absorption and distribution, with sepiolite consistently showing higher water release compared to agar gels, particularly in deeper layers.

The data acquired by means of NMR device allow the following further considerations:

in Noto calcarenite water penetrates up to 5 mm with sepiolite, while agar pushes the water penetration to 1–3 mm, ensuring a non-superficial cleaning action;

a very similar trend has been shown by Manciano sandstone: a smaller amount of water is absorbed with respect to Noto calcarenite, but around the same penetration depth is reached;

NMR data confirm the higher water release from Bresciani agar than from CTS agar;

for Black Bergamo limestone, only a superficial layer of water is released, with almost no penetration.

### 2.4. Water Absorbed by Stone Specimens Measured by Means of SUSI Probe

SUSI testing was carried out on stone specimens; to this aim a set of five specimens for each stone was moistened with the procedure already described.

**Figure 8:**
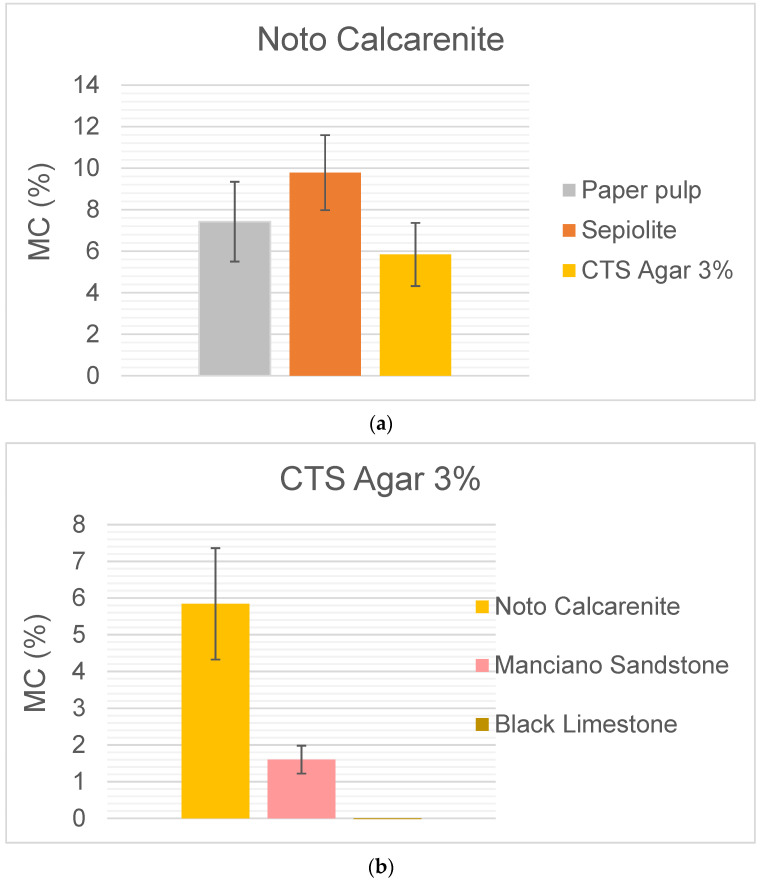

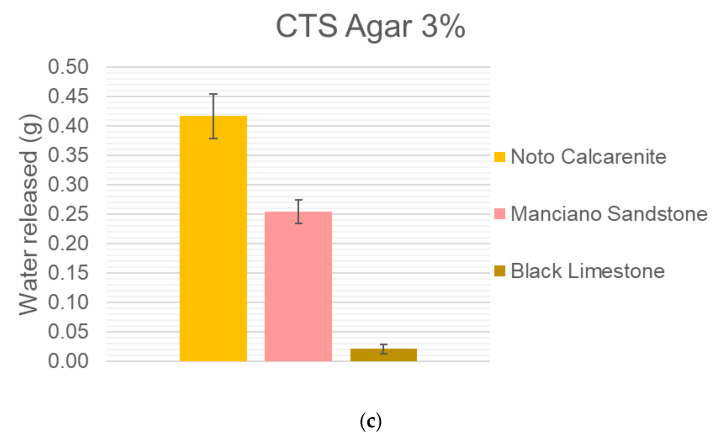
(**a**) Noto calcarenite Moisture content (MC%) after water release by traditional and CTS Agar 3%. (**b**) Comparison of the moisture content (MC%) released on the three different stones. (**c**) Comparison of the water released by CTS Agar 3% on the three different stones.

Then the MC % provided by the instrument was measured. The measures provided the following data shown in Figure 8a.

For water release by traditional pads and gels, the trend given by gravimetric measurements is confirmed, with release by CTS agar lower than that by traditional pads (around one third less)

The SUSI data indicate that, among the traditional pads, the one releasing more water is sepiolite; these data are different from those from gravimetric measures, where the pad releasing more water is paper pulp.

The water release trends measured by gravimetry and by SUSI are very similar, as shown in Figure 8b,c.

## 3. Conclusions

The results obtained highlight the different performances given by the tested cleaning systems on the various tested stone substrates. The following points summarize the main scientific results.

Traditional pads release a larger amount of water than gels, from 2 to 5 times, depending on the stone considered.

Paper pulp releases the greatest amount of water in any case: in the case of Noto calcarenite water released by agar gel (Bresciani 3%) is only 16.07% of the water released by paper pulp; in the case of Manciano sandstone the water released by Bresciani agar 3% rises to 31.09%. The comparison of sepiolite vs. Bresciani agar 3% indicates that the latter releases only 25.86% (Noto) and 39.73% (Manciano).

Bresciani agar released a larger amount of water with respect to the other tested agar gels (in the case of Noto stone, water release by Bresciani agar is 211% with respect to CTS agar 3%; in the case of Manciano sandstone the increase is around 127%).

The overall results advise conservators to choose any agar gel for cleaning very sensitive materials, such as murals, gypsum-based stuccoworks, or paper. The system can be improved through the use of Japanese paper, which can reduce water release by up to 75%.

Gravity does not influence the measures which can be carried out both in horizontal and in vertical geometry; hence, the results acquired in this study can be transferred to vertical surfaces on conservation sites.

Regarding possible future research plans, lithotypes should be chosen with an appropriate porosity range; very low porosity stones, such as Black Bergamo limestone, proved to be not reliable due to the high experimental errors.

It is important to note the low values of standard deviation for Noto and Manciano stones, assuring the data precision quality.

A possible list of errors includes the difficulties in correctly shaping a pad of the desired sizes using sepiolite and paper pulp. Moreover, as the shaping operation was not simple, it requires some time to be performed, during which the water release starts. The need for squeezing these kinds of traditional pads is a problem in reproducibility as well. Thus, experiments with both sepiolite and paper pulp are hardly reproducible.

On the contrary, agar gels can be cut in Petri dishes and allow a better reproducibility.

In the everyday life of conservators, a challenge is to match the pads’ correct range of porosity in order to have the most effective water release, for example, for desalination. In fact, to obtain the best desalination effect, the pads’ pores should compete with the stones’ porous system to induce the best outward suction of the solutions formed inside the pores. In attempting to do this, conservators prepare blends by mixing together paper pulp, sepiolite, sand, and gels. As part of this research, the authors prepared three different typologies of mixes (sepiolite, paper pulp, and sand, in various proportions), testing them on the stones used in the experimentation. Unfortunately, no specific trends as concerns water release have been individuated, hence the corresponding data are not presented. The authors suppose that a great number of variables, not easily controlled, influence the obtainment of reliable data; just to name a few, these mixes should be manually squeezed in order to reach the right consistency to be applied on the stone surface. At the moment the authors do not present a procedure to prepare a reproducible mix. To predict and to explain the performance of these mixes is an open issue and a challenge for future works for the scientific community.

## 4. Materials and Methods

### 4.1. Reference Stones

The lithotypes used are Noto calcarenite, Manciano sandstone, and Bergamo black limestone [32]; these materials are classified as sedimentary rocks and they have been chosen because of their different porous systems, both in terms of the total percentage of porosity and pore size distribution. Moreover, they were part of a previous research program on the common topic of gel water transfer phenomena on stone materials [14]; for these reasons they have been considered as a reference.

### 4.2. Preparation and Application of Water-Releasing Materials

Traditional pads were made with paper pulp and sepiolite, two systems allowing a different water distribution in the microstructure.

As paper pulp, Arbocel^®^ BC 1000 has been used, chosen because of its widespread presence in the market; the product is capable of mixing with an amount of water equal to 4/5 times its weight [33].

Sepiolite [34] can be blended with a large amount of water while avoiding excessive swelling.

Very often and especially in the latter case, traditional pads are used on stone surfaces by inserting a Japanese paper sheet in order to allow a faster and more complete removal of the pads, avoiding any residues on the stone surface [35].

The optimization of each pad and gel application is part of the research plan; it has been designed considering the following requirements: a good adhesion between pad (or gel) and stone surface; reproducible size and thickness of the pads or gels; reproducible water content in each pad or gel, used on a set of 5 identical 5 × 5 × 2 stone specimens; minimization of any experimental error and optimization of a reproducible procedure.

Pads and gels of 5 × 5 × 1 cm were prepared, so that they could be perfectly superimposed on the stone specimen polished surface, each with an equal “reservoir” 1 cm thick. Traditional pads were placed directly on the stone surface while gels were placed inside a Petri dish, then cut and positioned on the stone surface. Each contact time between traditional pads/gels and stones was 30 min. During the measuring test the pad (or the gel) was covered with a polyethylene film to avoid water evaporation. After this period, the pad or gel was delicately removed, paying attention not to leave any residue, then the stone specimen was immediately weighed.

To study the water release kinetics of gels during their application on the stone surface, both gels and stone specimens were weighed at specified timings (every 5 min during the first 30 min, then after 60 min).

In the following, each procedure is detailed.

Paper pulp pads were prepared with the ratio of paper:water reported in Table 4. The paper pulp pads were applied on the surface and shaped till reaching a uniform 1 cm thickness, then weighed. When required, Japanese paper was inserted. The first weight was used as a reference for the whole series of five specimens, so that the following paper pulp pads were prepared with exactly the same weight. After 30 min, the polyethylene film and the pad were removed and the specimen was immediately weighed. At the end of the procedure, the same specimens were put in an oven at 60 °C to be prepared for the next steps.

Sepiolite pulp pads were prepared with the ratio of sepiolite:water reported in Table 4; then the procedure was identical to the one used for paper pulp pads.

Following a previous experimentation attempting to remove soluble salts from a plaster dated back to the XV century [19], the authors tested three different mixes composed of sepiolite, paper pulp, and sand to match the different microstructures of the pads, hence having different water transport phenomena and water releases. The three mixes tested are described in Table 4. Conservators blend different materials in order to match the most appropriate pad and optimize the soiling extraction capability and the water release [36]. Both of these physical phenomena mostly depend on the porous system present in the pad. The operation attempts to optimize the cleaning effects especially during desalination. Even in this case, water release has never been measured. Hence, the present study tested three mixed materials, obtained by blending paper pulp, sepiolite, and sand (see Table 4).

**Figure 9:**
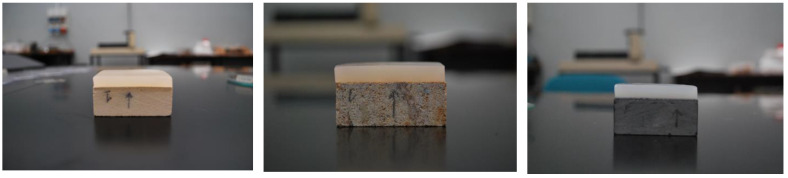
From left to right: Noto calcarenite, Manciano sandstone, Bergamo black limestone with rigid Bresciani agar gel 3%.

It has been proven that the different agar products available in the market are slightly different in their composition and properties, depending on natural variability but also on different extraction techniques from the raw materials [37]. For what concerns the Italian and, more generally, the European market, both CTS (CTS s.r.l., Monza, Italy) and Bresciani (Bresciani s.r.l., Milano, Italy) products are the most widespread. Moreover, they were compared with a Sigma product (A7002, Sigma-Aldrich, St. Louis, MO, USA). Agar powders (CTS AgarArt; Bresciani; Sigma) were mixed with water in the specified concentration (3%); for Bresciani powder, gels with concentrations of 4 and 5% were prepared as well to study the rigid gels’ performance as a function of increasing concentration. Mixes were heated in a microwave oven (700 W–2 min); then the sol produced was re-heated in the same condition. Conservators heat the agar gels twice in order to improve their rheological and chromatic features: agar gels heated twice loose the yellowish color obtained after the first heating, and their transparency increases. Then, the sol was poured into a Petri dish up to a thickness of 1 cm, cooled at room temperature, and sized to 5 × 5 cm. Agar gels were used on three stones (Figure 9), either as warm fluid (around 40–45 °C) or as rigid gels at room T.

**Figure 10:**
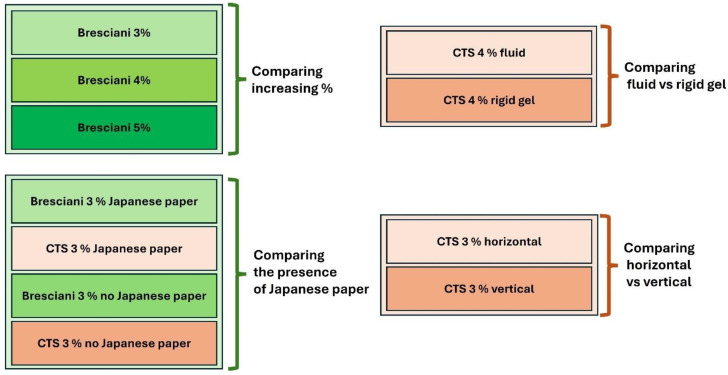
Synoptic scheme of the overall comparison among gels.

The tested agar gels were studied and the comparisons are schematized in Figure 10.

It is possible to see from Figure 10 that the testing procedure included another two points:

1. comparison between the water released by an agar gel in a warm fluid condition (38/40 °C) and the same gel rigid at room temperature;
2. to answer the research question regarding any possible differences among all the tested materials presented here, with a horizontal or vertical position, so that the influence of gravity force can be induced and the geometry usually present in building conservation sites can be studied.

### 4.3. Study Methods

#### 4.3.1. Procedure to Carry Out the Gravimetric Measures on Stone Specimens

The European standard EN 15801:2009 (Conservation of cultural property—Determination of water absorption by capillarity) inspired the method used for the gravimetric measures on the stone specimens [38]. The standard suggests the use of a pad made with filter paper on which a stone specimen is placed. In the present paper, two variations have been introduced to fulfil the needs of the gravimetric measures:

(a) to substitute the filter paper with the specific material under testing (sepiolite, paper pulp, and gels);
(b) to invert the geometry of the measure: here we used the pad positioned on the specimen (see Figure 9).

Measures were carried out on a set of 5 specimens per lithotype, sized 5 × 5 × 2 cm.

Specimens were prepared by polishing one of the two major faces sized 5 × 5 cm (Struers polishing equipment with the following parameters: 60 s, 5 N force, 150 rpm with water, P32021 FEPA abrasive paper). Then, specimens were washed, brushed, immersed in deionized water for 30 min, and put in an oven (60 °C for 24 h) for desiccation until reaching a constant weight.

Measures obtained from 5 specimens were used calculate the average weight and the standard deviation.

#### 4.3.2. Evanescent Field Dielectrometry (EFD)

**Figure 11:**
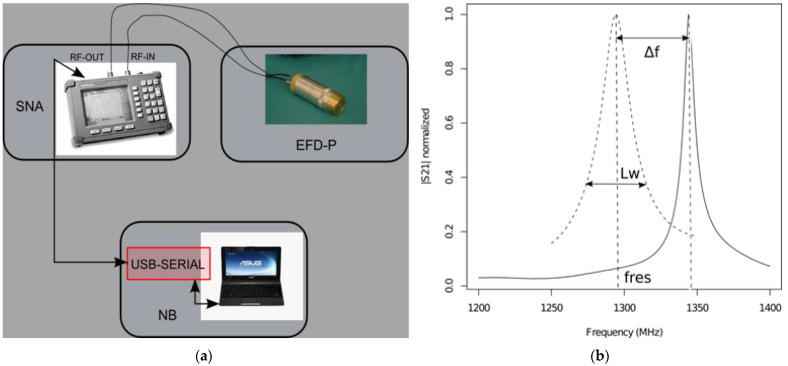
(**a**) SUSI© system consisting of: scalar network analyzer (SNA), notebook (NB), and EFD-P probe; (**b**) the solid line is the frequency response for the unloaded resonant probe, the dotted line is the behavior for the probe in contact with the material under investigation (humidity and salt quantity not specified).

In principle, the EFD technique uses the difference in the measures between the dielectric contrast of the water and the penetrated host material in the microwave frequency spectrum; the instrument measures the permittivity (dielectric constant and loss factor) by a resonant method [39]. The EFD system used here (called SUSI©) consists of a resonant probe operating in the microwave frequency range (1–1.5 GHz) of a scalar network analyzer (SNA) for measuring the probe frequency response and a laptop that drives the device by dedicated software and provides the diagnostics results (humidity content and salinity index) from the electrical parameters (Figure 11a). Water has a dielectric constant value of about 80; stone materials used in architecture (bricks, plaster, mortar, stone) have a dielectric constant of about 3–5; this strong difference in values generates a dielectric contrast between the water and the material, which is used to carry out a measurement of the moisture content and the soluble salts contained in the material up to a depth of 2 cm from the surface.

The electrical parameters of interest of the resonant probe response are the resonance frequency fres and the quality factor Q (or resonance bandwidth Lw) shown in Figure 11b. Δfr corresponds to the resonant frequency shift of the probe between the loaded behavior, when in contact with the material under test (fres), and for an unloaded probe (in air) (fo). These resonant parameters are related to the diagnostics parameters moisture content (MC) and salinity index (SI) [40,41,42]. In the first instance, the frequency shift is related to the MC while the spreading of the bandwidth is due to the combination of the dielectric losses and conductivity losses in the microwave range, both related to the presence of electrolytes in solution (i.e., presence of salts). The MC of the examined material may be related to Δfr, according to the simplified equation shown in references [40,41,42], where a calibration procedure is presented for the determination of the correlation constants between electrical parameters and diagnostics parameters (MC and SI). The humidity content that can be investigated is in the range 0% to 20% (on a dry basis), and for the salinity index it is from 1 to 10. The SI is a semi-empirical parameter, determined by an experimentation on plaster samples with different salinity to obtain a calibration curve [43].

#### 4.3.3. Portable Nuclear Magnetic Resonance

Gels obtained from different commercial agar powders show differences in porosity. An investigation conducted using portable nuclear magnetic resonance (NMR) illustrates the mobility of water within the polysaccharide network of agar gels and demonstrates that the mobility of water molecules in the pores strongly depends on the concentration of polysaccharide [37]. As the double helices and their interconnections increase, the mobility and the values of free water in the gel decrease.

Additionally, another key factor in the absorption, distribution, and penetration of water into the stone depends not only on the physico-chemical properties of the gel but is also influenced by the porosimetric characteristics of the porous material itself. ¹H NMR depth profiles are a powerful tool for assessing water absorption in porous materials. This technique involves the use of nuclear magnetic resonance (NMR) to measure the presence and distribution of hydrogen atoms (¹H) within a sample, which is indicative of water content [44]. In this study, ^1^H NMR depth profiles were acquired to examine the amount of water released into three different stones with varying porous structures, as well as the distribution and penetration depth of water absorbed by the stones. All measurements were carried out 30 min after the application of gels at 13.62 MHz using a portable NMR device from Bruker Biospin connected to a single-sided sensor developed by RWTH Aachen University, Germany [45]. This sensor was mounted on a lift that moved relative to the sample, allowing for precise micrometric positioning of the excited slice, enabling us to acquire ^1^H depth profiles. The intensity of the ^1^H depth profiles recorded on stone samples after the application of gels was determined by summing the first four echoes collected using a Carr–Purcell–Meiboom–Gill (CPMG) pulse sequence (2t of 57 µs, nominal resolution of 0.06 mm, with 0.2 mm steps). All profiles were acquired by scanning the specimen to a maximum depth of 5 mm.

## Figures and Tables

**Table 1:** Lithotype, % porosity, pore distribution, and pore diameter main range.

Lithotype	Porosity (%)	Pore Distribution	Pore Diameter Main Range (μm)
Noto calcarenite	36.2	unimodal	0.5–5.0
Manciano sandstone	10.7	unimodal	0.01–5.0
Bergamo black limestone	0.5	unimodal	1.0–4.0

**Table 2:** Percentage porosity distribution in the three stones under test.

Lithotype	Noto	Manciano	Bergamo
Megapores (%)	12.2	25.9	12.8
Macropores (%)	86.8	67.0	67.4
Mesopores (%)	1.0	7.1	19.7

**Table 3:** Water release by CTS Agar 3%; water absorption capillarity coefficient (N.D. = not detected).

Lithotype	CA [(Kg/m^2^)/sec^^1/2^]
Noto Calcarenite	3.80 × 10^−3^
Manciano Sandstone	2.10 × 10^−3^
Black Limestone	N.D.

**Table 4:** Tested Mixes. Composition of the traditional pads used.

Pad	Thickener	Water (mL)	Arbocel BC 1000 (g)	Sepiolite (g)	Sand (g)
Paper pulp	Arbocel BC 1000	200	35	/	/
Sepiolite	Sepiolite CTS	200	/	120	/
Mix 1	Arbocel + sepiolite	200	26	30	/
Mix 2	Arbocel + sepiolite + sand	200	26	30	15
Mix 3	Arbocel + sepiolite + sand	200	17	60	15

## Data Availability

The data presented in this study are available on request from the corresponding author.
